# Molecular bases of K^+^ secretory cells in the inner ear: shared and distinct features between birds and mammals

**DOI:** 10.1038/srep34203

**Published:** 2016-09-29

**Authors:** Viviane Wilms, Christine Köppl, Chris Söffgen, Anna-Maria Hartmann, Hans Gerd Nothwang

**Affiliations:** 1 Neurogenetics group, Cluster of Excellence “Hearing4All”, School of Medicine and Health Sciences, Carl von Ossietzky University Oldenburg, 26111 Oldenburg, Germany; 2 Cochlea and Auditory Brainstem Physiology, Cluster of Excellence “Hearing4All”, School of Medicine and Health Sciences, Carl von Ossietzky University Oldenburg, 26111 Oldenburg, Germany; 3 Research Center for Neurosensory Sciences, Carl von Ossietzky University Oldenburg, 26111 Oldenburg, Germany; 4 Systematics and Evolutionary Biology Group, Institute for Biology and Environmental Sciences, Carl von Ossietzky University, 26111 Oldenburg, Germany

## Abstract

In the cochlea, mammals maintain a uniquely high endolymphatic potential (EP), which is not observed in other vertebrate groups. However, a high [K^+^] is always present in the inner ear endolymph. Here, we show that Kir4.1, which is required in the mammalian stria vascularis to generate the highly positive EP, is absent in the functionally equivalent avian tegmentum vasculosum. In contrast, the molecular repertoire required for K^+^ secretion, specifically NKCC1, KCNQ1, KCNE1, BSND and CLC-K, is shared between the tegmentum vasculosum, the vestibular dark cells and the marginal cells of the stria vascularis. We further show that in barn owls, the tegmentum vasculosum is enlarged and a higher EP (~+34 mV) maintained, compared to other birds. Our data suggest that both the tegmentum vasculosum and the stratified stria vascularis evolved from an ancestral vestibular epithelium that already featured the major cell types of the auditory epithelia. Genetic recruitment of Kir4.1 specifically to strial melanocytes was then a crucial step in mammalian evolution enabling an increase in the cochlear EP. An increased EP may be related to high-frequency hearing, as this is a hallmark of barn owls among birds and mammals among amniotes.

Mechanotransduction in the vertebrate inner ear, both auditory and vestibular, is mediated by K^+^ and Ca^2+^ influx into hair cells. The apical part of hair cells, their mechanosensitive hair bundle, is bathed in a very unusual extracellular fluid, the endolymph of scala media^1^,^2^. This fluid resembles in its ionic composition an intracellular milieu, as it features a high concentration of K^+^ and a low concentration of Na^+^ (elasmobranchs^3^, frogs^4^, turtles^4^, lizards^3^,^4^, birds^4^, mammals^1^). Furthermore, this endolymph shows a positive potential (endolymphatic potential, EP) compared to other extracellular spaces^5^. The EP provides an enhanced electrical gradient for positively charged ions to flow into the hair cells during stimulation when transduction channels open. The ionic composition, with K^+^ as the dominant cation, then determines that the transduction current is mostly carried by K^+^, with Ca^2+^ being the second most important contributor^6^. Since the driving force for K^+^ to enter hair cells is predominantly electrical, the EP critically contributes to the exquisite sensitivity of hair-cell mechanotransduction^2^.

Although the basic mechanism of hair-cell mediated mechanotransduction is conserved between all vertebrates, an important difference concerns the extent of the positive EP. Frogs, turtles, snakes, lizards and crocodilians typically maintain only +2 to +7 mV^5^. Birds show somewhat higher EP levels, typically between +10 and +20 mV^7^,^8^. Finally, high values are observed in therian mammals with an EP of typically +80 to +90 mV^1^,^5^.

The large difference in the EP between non-mammalian and mammalian tetrapods is paralleled by changes in the secretory epithelia that generate the endolymph in scala media. In non-mammalian tetrapods, this epithelium overlies the basilar papilla (BP) and separates scala media and scala vestibuli^9^. In birds, it is composed of a single layer of cells that form a series of folds into the scala media and has been termed tegmentum vasculosum^9^. In mammals, the functionally equivalent structure is situated in the lateral cochlear wall and composed of two tissues. The spiral ligament contains five distinct types of fibrocytes and connective tissue, whereas the epithelial stria vascularis is a stratified tissue comprising marginal, intermediate, and basal cells^2^.

In the mammalian cochlea, the molecular and cellular mechanisms generating the endolymph and the EP have been investigated in great detail^1^,^2^. In a first step, K^+^ is taken up by fibrocytes of the spiral ligament and transported to intermediate and basal cells of the stria vascularis, via a syncytial gap-junction network. Intermediate cells then release K^+^ into the intrastrial space, from where it is taken up by marginal cells. These cells form a single layer and release K^+^ at their apical surface into scala media. The two-step process ultimately results in an EP of +80 to +90 mV and approximately 150 mM [K^+^] in scala media^1^,^2^. Recent years have witnessed the identification of major molecular players in this K^+^ secretion route in the lateral cochlear wall. It comprises connexins 26 and 30, the plasma membrane transporters Na^+^-K^+^-ATPase and NKCC1, the K^+^ channels Kir4.1, Kir 5.1, KCNQ1, and KCNE1, as well as the Cl^-^ channel proteins CLC-Ka, CLC-Kb, and Barttin^1^,^2^. Mutation in most of these genes is associated with deafness in humans or mice^10^,^11^.

In summary, there are marked differences concerning both the EP and the cellular basis of the generation of the endolymph between mammals and other tetrapods. This raises two important questions regarding proximate and ultimate causes^12^: (1) what evolutionary changes caused the emergence of the high mammalian EP (“how” question) and (2) what are the selective pressures that may have led to the exceptionally high EP in mammals (“why” question)? Here we addressed these questions by performing molecular and electrophysiological studies in birds that show a more moderate but still significantly enhanced EP. We chose two avian species with distinct auditory capabilities, the chicken as an auditory generalist and the barn owl as a known high-frequency specialist^13^.

## Results

An unusually highly positive EP is a hallmark of the mammalian cochlear endolymph. To investigate whether this uniquely high EP is associated with evolutionary differences in the molecular underpinnings, we decided to characterize in the chicken the expression pattern of genes that are known to be involved in mammals. For our study, 14 mammalian genes were selected: *ATP1A1*, *ATP1B1, ATP1A2*, *ATP1A3 encoding* Na^+^-K^+^-ATPase subunits, *BSND, CLCNKA, and CLCNKB,* encoding Cl^−^ channel subunits, *KCNJ10*, *KCNJ16*, *KCNQ1*, *KCNE1* encoding K^+^-channels, and the gap junction genes *GJB2* and *GJB6*, and *SLC12A2,* encoding the Na^+^-K^+^-Cl^−^ cotransporter NKCC1. For most of them, chicken orthologs could be identified in *GenBank* or *ensemble.* The only exceptions were the three genes encoding Cl^−^-channel proteins, as no reliable database entries were detected when we initiated this study.

### Phylogenetic analysis of CLC-K and BSND

**Figure 1:**
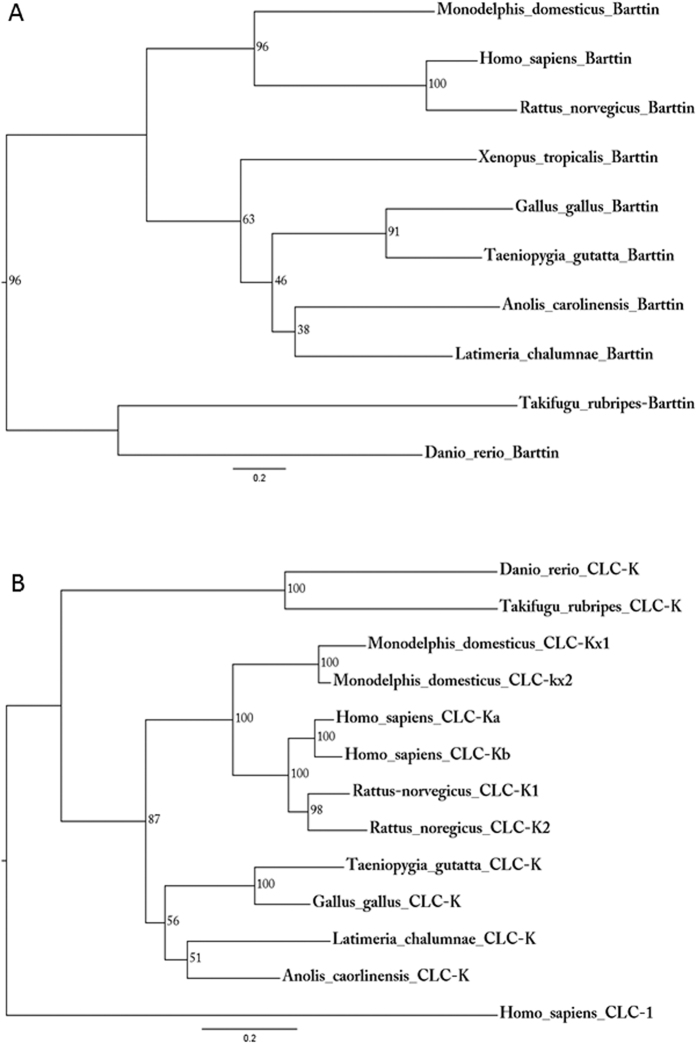
Phylogenetic analyses of Barttin and CLC-K. Phylogenetic trees of vertebrate Barttin (**A**) and CLC-K (**B**) were constructed using maximum-likelihood analysis (PhyML, model: Blosum62) with 1,000 replicates. Only bootstrap scores >50% are indicated. The accession numbers of all proteins are listed in Tables 1 and 2.

To identify the chicken orthologs of *BSND* and *CLC-K* channel proteins, phylogenetic analyses were performed using available protein sequences from different vertebrate species (Tables 1 and 2). For Barttin, a single protein was identified in all vertebrate species (Fig. 1A). The CLC chloride channel family comprises three subfamilies: CLC-1, -2 and CLC-K; CLC3, -4, -5, and CLC-6 and CLC7. Phylogenetical analyses of CLC-K rooted with the human CLC-1 sequence indicate that all vertebrate groups except for amphibians (*Xenopus laevis*) contain at least one CLC-K protein sequence (Fig. 1B). Independent gene duplication events resulted in two paralogous CLC-K protein sequences in *Homo sapiens, Rattus norvegicus* and *Monodelphis domesticus*. These channels were named CLC-Ka and CLC-Kb in human, CLC-K1 and CLC-K2 in rat and CLC-Kx1 and CLC-Kx2 in *Monodelphis domesticus*. Similar conclusions were obtained in a previous, more limited, analysis which also revealed that the amino acid sequence identity between the paralogous CLC-K proteins of human and rat (*Homo sapiens* CLC-Ka and CLC-Kb, *Rattus norvegicus* CLC-K1 and CLC-K2) is larger than between CLC-K proteins across species (see also Table 3)^14^.

### RNA *in situ* hybridization in the tegmentum vasculosum

To study the expression pattern of the genes in the avian inner ear, RNA *in situ* hybridization with DIG labeled antisense probes was performed on cross sections of the cochlea isolated from chickens aged 12 to 17 days posthatching (P12 to P17). At this age, the chicken EP is mature^15^.

#### Plasma membrane transporters

**Figure 2:**
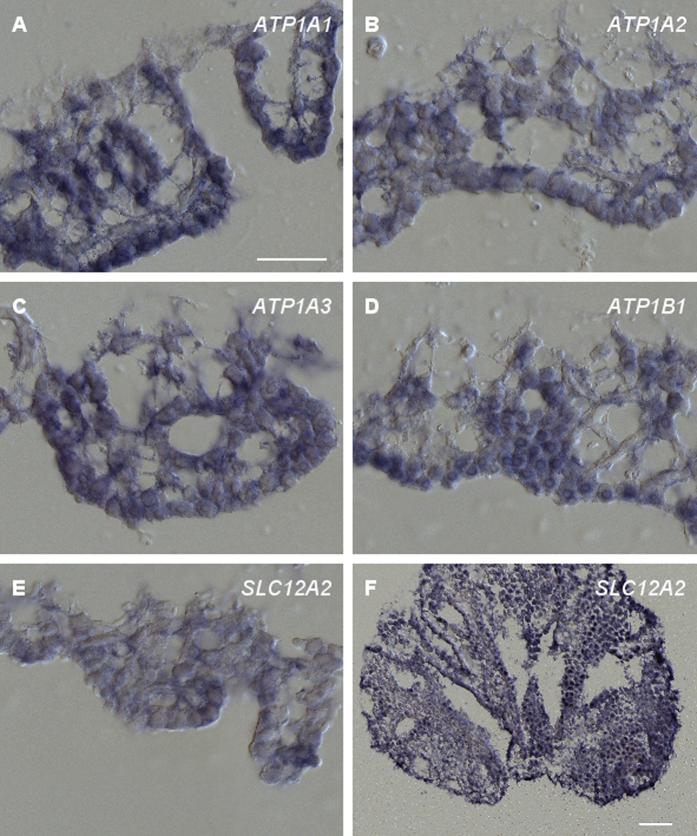
RNA *in situ* hybridization of plasma membrane transporter-related probes on cross sections of the tegmentum vasculosum. RNA *in situ* hybridization of cross sections through the chicken tegmentum vasculosum (**A**–**E**) or barn owl (**F**). Sections were hybridized with DIG-labeled cRNA probes, as given in the images. All probes hybridized throughout the epithelium. Representative results from at least 3 independent hybridization experiments are shown. (**A**–**E**), scale bar 50 μm; F, scale bar 100 μm.

We first focused on members of the Na^+^, K^+^-ATPase, as they are part of the K^+^ uptake apparatus in fibrocytes of the spiral ligament and marginal cells of the stria vascularis^1^,^11^. *ATP1A1,* encoding the Na^+^, K^+^-ATPase α1 was shown to be the sole alpha Na^+^, K^+^-ATPase isoform present in the stria vascularis in adult mice^16^ and rats^17^, and in the spiral ligament of adult mice^16^. During the first month of life, the mouse spiral ligament expresses in addition *ATP1A2* and *ATP1A3*^16^. We therefore analyzed the expression pattern of *ATP1A1, ATP1A2* and *ATP1A3* in the chicken inner ear. All three genes were expressed throughout the tegmentum vasculosum (Fig. 2A–C). We could not distinguish between light cells and dark cells in the tegmentum vasculosum, a distinction mainly made in electron microscopy studies through the different electron density of the cells (osmiophilic dark cells, osmiophobic light cells) or using enzymatic reactions. Cytological differences, such as the apical microvilli or the extended plasma membrane infoldings throughout the sides and the basolateral part of dark cells^9^, were not discernable, likely due to the rather harsh RNA *in situ* hybridization conditions.

The expression pattern of the three genes in the tegmentum vasculosum resembled that of fibrocytes in the spiral ligament. We therefore included in our analysis *ATP1B1,* encoding the Na^+^, K^+^-ATPase β1. This isoform is also strongly expressed in mammalian fibrocytes but only poorly in the stria vascularis^16^,^17^. Again, clear expression of the gene was observed throughout the tegmentum vasculosum (Fig. 2D). Taken together, all four subunits of the Na^+^, K^+^-ATPase were expressed in the tegmentum vasculosum with no obvious difference in their expression pattern.

The Na^+^, K^+^, Cl^−^-cotransporter NKCC1 mediates cellular uptake of K^+^ and is present in mammals in both fibrocytes and in marginal cells of the lateral wall. In the chicken inner ear, expression of the corresponding gene *SLC12A2* was observed throughout the tegmentum vasculosum (Fig. 2E). Previous pharmacological experiments in pigeon had suggested that NKCC1 is not involved in the generation of the EP in the avian scala media^18^. We therefore analyzed expression of *SLC12A2* in another bird, the barn owl. Again, we observed clear expression in the tegmentum vasculosum (Fig. 2F). This result confirmed that NKCC1 is part of the molecular repertoire of the avian tegmentum vasculosum.

#### Plasma membrane channels

**Figure 3:**
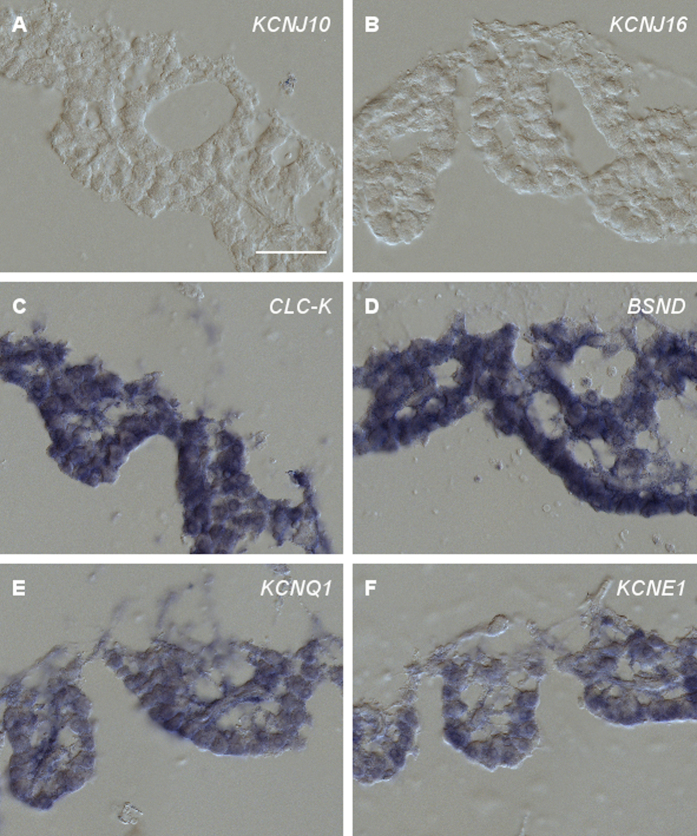
RNA *in situ* hybridization of plasma membrane channel-related probes on cross sections of the tegmentum vasculosum. RNA *in situ* hybridization of cross sections through the chicken tegmentum vasculosum (**A**–**F**). Sections were hybridized with DIG-labeled cRNA probes, as given in the images. Most probes hybridized throughout the epithelium with no distinction between dark and light cells. The only exceptions were *KCNJ10* and *KCNJ16*, which gave no positive signal (**A**,**B**). Representative results from at least 3 independent hybridization experiments are shown. Scale bar, 50 μm.

Two *KCNJ* genes, *KCNJ10* and *KCNJ16*, play important roles in the generation of the endolymph. *KCNJ16*, encoding the inwardly rectifying K^+^ channel Kir5.1, is expressed in fibrocytes of the spiral ligament where it likely controls cochlear K^+^ circulation^11^. Kir4.1, encoded by *KCNJ10*, resides in the apical membrane of intermediate cells in the stria vascularis and is crucial for the generation of the EP^19^. In the chicken inner ear, neither the tegmentum vasculosum nor any other tissue was labeled using *KCNJ10* and *KCNJ16 in situ* probes (Fig. 3A,B). Labeling of neural populations such as the Nucleus reticularis lateralis in coronal sections of the brain and of cross sections of the kidney with the *KCNJ10* and *KCNJ16* probes, respectively, demonstrated that the absence of labeling in the inner ear was not due to inappropriate probes (data not shown). Thus, neither inwardly rectifying K^+^ channels Kir4.1 nor Kir5.1 are present in the chicken tegmentum vasculosum. The absence of Kir5.1 which resides in fibrocytes of the mammalian spiral ligament is in agreement with the lack of such a tissue in the avian cochlea. As the barn owl exhibits an unusually high EP (see below), we tested the expression of *KCNJ10* also in this bird. Again, no expression was noted in the tegmentum vasculosum (data not shown).

The CLC-K channel and its accessory protein BSND reside in the basolateral membrane of strial marginal cells. They likely provide an efflux system to recycle Cl^−^ back to the intrastrial space, which is required for sustained NKCC1 activity^11^. Both genes were expressed throughout the tegmentum vasculosum (Fig. 3C,D).

Finally, the K^+^ channel proteins KvLQT1and IsK form a complex in the apical membrane of strial marginal cells that mediates final secretion of K^+^ to the endolymph. Both *KCNQ1* and *KCNE1,* encoding KvLQT1 and IsK, respectively, were expressed throughout the tegmentum vasculosum (Fig. 3E,F).

### RNA *in situ* hybridization in the Crista ampullaris

**Figure 4:**
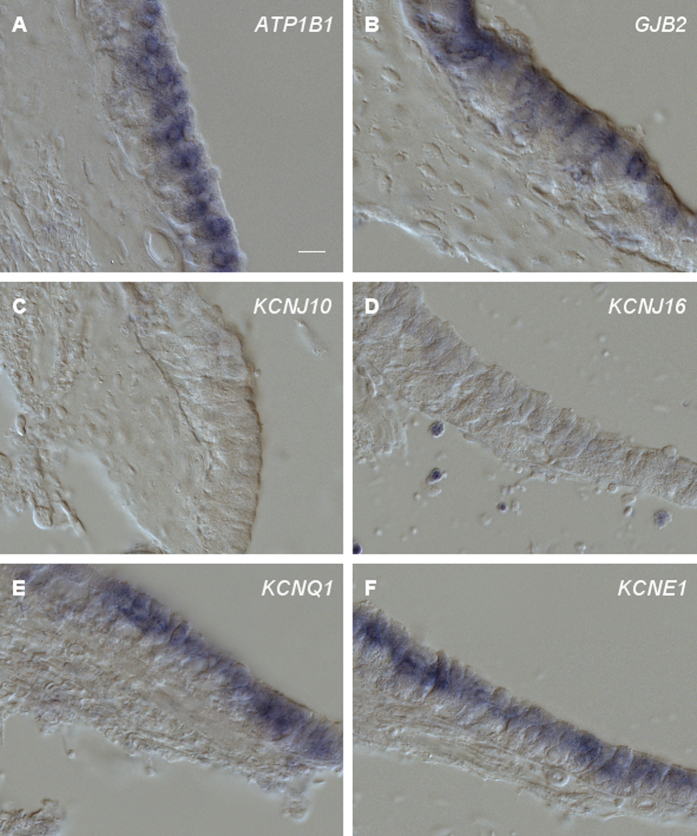
RNA *in situ* hybridization of probes on cross sections of the crista ampullaris. RNA *in situ* hybridization of cross sections through the chicken crista ampullaris (**A**–**F**). Sections were hybridized with DIG-labeled cRNA probes. *ATPB1, GJB2, KCNQ1,* or *KCNE1* derived probes hybridized to vestibular dark cells flanking the sensory epithelium, whereas *KCNJ10* and *KCNJ16* gave no positive signals (**C**,**D**). Representative results from at least 3 independent hybridization experiments are shown. Scale bar, 10 μm.

Both the auditory and the vestibular hair-cell organs of vertebrates are housed within the inner-ear labyrinth. Similar to their auditory counterparts, vestibular hair cells are bathed in endolymph. This vestibular endolymph displays a high concentration of K^+^ and an endovestibular potential of +1 mV in the semicircular canals^20^,^21^, similar to the endolymph in the scala media of non-mammalian tetrapods. In the ampullae of the three semicircular canals, endolymph is believed to be generated by vestibular dark cells flanking the neurosensory epithelium^11^,^21^. To examine whether these vestibular dark cells and the tegmentum vasculosum share critical molecular components, we also analyzed the expression of *ATPA1, ATPB1, KCNJ10, KCNJ16, KCNQ1, and KCNE1,* in the epithelia of the ampullae. In addition, we included *GJB2* and *GJB6,* which were previously shown to be expressed in the tegmentum vasculosum^22^. Probes against *ATPA1, ATPB1, KCNQ1, KCNE1, GJB2* and *GJB6* clearly labeled the vestibular dark cells (Fig. 4A,B,E,F), shown for *ATPB1, GJB2, KCNQ1, KCNE1*). In contrast, no expression of *KCNJ10* and *KCNJ16* was observed in the crista (Fig. 4C,D). Thus, tegmentum vasculosum and the vestibular dark cells of the ampulla show a congruent expression pattern of genes involved in the generation of endolymph.

### Tegmentum vasculosum and EP in the barn owl

**Figure 5:**
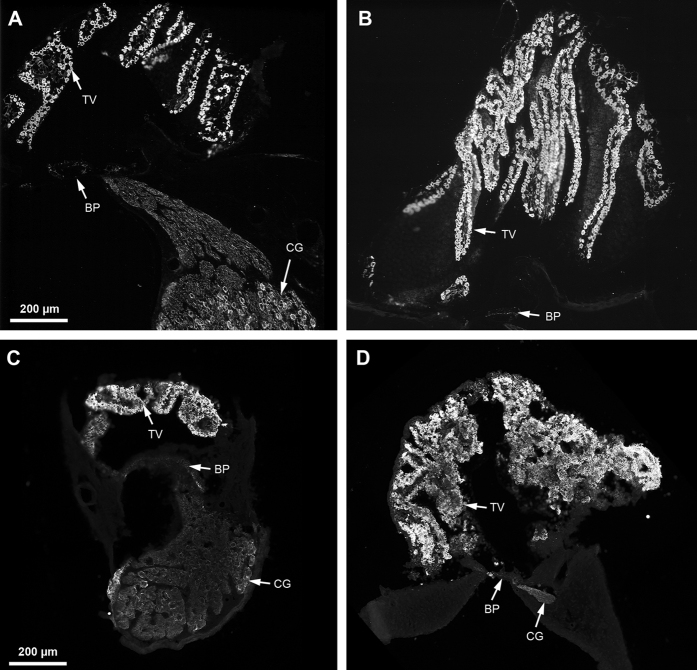
Tegmentum vasculosum of the barn owl and the chicken. Example cross sections of a barn owl and chicken basilar papilla at two locations: (**A**) Barn owl basilar papilla 29% from the apical end, corresponding to the tonotopic location of 3 kHz. (**B**) Barn owl basilar papilla 67% from the apical end, corresponding to 8 kHz. (**C**) Chicken basilar papilla 30% from the apical end, corresponding to 330 Hz. (**D**) Chicken basilar papilla 70% from the apical end, corresponding to 3 kHz. Sections show the immunofluorescent signal for Na^+^, K^+^ ATPase which was brightest in the tegmentum vasculosum (TV), and fainter in the neural elements of the basilar papilla (BP), cochlear ganglion (CG) and connecting nerve fibers. Note the many-folded structure of the tegmentum vasculosum in the barn owl and its large size relative to the basilar papilla and to the tegmentum vasculosum of chicken, especially at the more basal, high-frequency location (**B**,**D**).

The highly positive EP in mammals may be associated with high frequency hearing, as hearing >10 kHz is a unique feature of therian mammals^23^. Among birds, barn owls show a unique extension of the sensitive hearing range up to 12 kHz^13^,^24^. We therefore decided to investigate the tegmentum vasculosum and the EP in barn owls, to examine whether they evolved unique features. Labeling of the tegmentum vasculosum using immunohistochemistry against Na^+^, K^+^ ATPase revealed that the tegmentum vasculosum is thicker and more folded than in other birds so far investigated, e.g. chicken (Fig. 5). The enlargement is most prominent in the basal regions of the basilar papilla (Fig. 5B) where high frequency sounds are transduced^13^.

**Figure 6:**
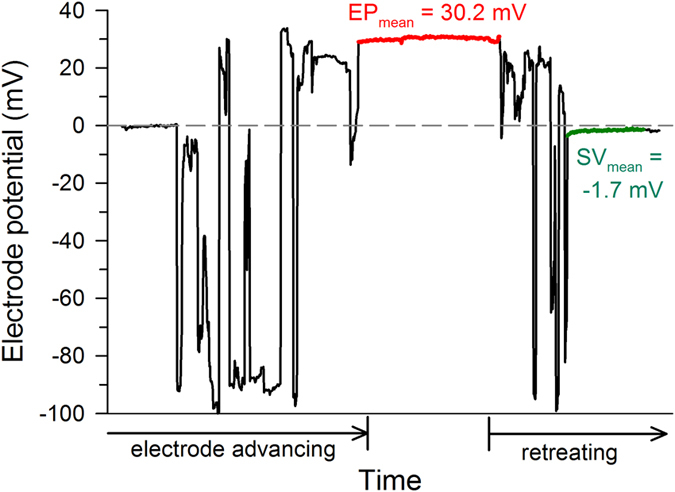
Example of an EP recording from the barn owl. The recorded potential is shown as a function of time. At the start of recording, the electrode potential was zeroed (dashed reference line) and then advanced in 10 μm steps at irregular intervals. Note that the graph does not show electrode position (depth), but time. Large negative potentials indicated that cells of the tegmentum vasculosum were entered. There were typically several large potential jumps over a distance of several hundred μm before a stable positive potential (here shown in red) could be recorded. Upon reversing the electrode movement, the potential returned to very close to baseline (here shown in green).

Measurements of the EP were obtained from 5 ears of 5 chickens and from 7 ears of 4 barn owls. The EP appeared as a sudden jump in the recorded potential to positive values, associated with a forward step of the electrode. Fig. 6 shows a typical recording. EP values for the chicken ranged from 13.5 to 17.5 mV (median 14.3 mV), confirming previous measurements^7^,^25^. Values for the barn owl ranged from 30.1 to 44.3 mV (median 33.8 mV). The EP of the barn owl was thus significantly higher than that of the chicken (Mann-Whitney U-Test, p = 0.003).

## Discussion

The auditory systems of extant birds and mammals reflect 300 million years of parallel evolution^26^. This has resulted in marked differences such as a coiled cochlea, a three-ossicle middle ear, an increased EP, prestin-based electromotility, and an extended hearing range in mammals^26^,^27^,^28^. The evolutionary processes and selective pressure(s) for many of the mammalian-specific features remain enigmatic. Here, we identified shared and distinct molecular features for the generation of the endolymph between birds and mammals. Our data pinpoint recruitment of Kir4.1 to the mammalian secretory epithelium as a crucial evolutionary event on the molecular level, enabling the generation of a highly positive EP. Otherwise, the molecular toolkit for K^+^ secretion into the auditory endolymph of the two vertebrate groups is rather similar. Finally, we demonstrate that the barn owl, a hearing specialist with an extended high-frequency hearing range among birds, has the highest EP measured in any non-mammalian animal. Thus, an increased EP in this specialized bird and in mammals generally correlates with the evolution of higher-frequency hearing.

### Proximate cause: Evolution of the K^+^ secretion apparatus

To answer the question what in tetrapod evolution caused the emergence of the high mammalian EP, we characterized the molecular repertoire underlying the generation of the more moderate but still significantly positive endocochlear potential in the avian scala media. This analysis revealed that two of the tested genes, *KCNJ10 and KCNJ16*, were not expressed in the tegmentum vasculosum. Mutations in *KCNJ10* cause deafness in mice^29^ and humans^30^. The encoded protein Kir4.1 is expressed in the apical membrane of intermediate cells of the stria vascularis and is thought to be a molecular key to the generation of the highly positive mammalian EP^31^. The apical membrane of intermediate cells is separated from the basolateral membranes of the marginal cells by an electrically isolated intrastrial space. The fluid of this space exhibits a low K^+^ concentration (1–2 mM) whereas the cytosol of intermediate cells has the usual high K^+^ concentration (140–150 mM)^32^,^33^,^34^. This constellation allows Kir4.1 to generate approximately +100 to +110 mV transmembrane K^+^-diffusion potential^2^,^19^,^32^,^35^ which is the basis for the high mammalian EP. The lack of this gene in the avian tegmentum vasculosum is in full agreement with such a role, as the avian EP is considerably lower. Furthermore, Kir4.1 is also absent in the mammalian vestibular system^31^, which also exhibits a low positive endovestibular potential of +1 mV^20^. Importantly, vestibular dark cells closely resemble marginal cells with respect to the expression and localization of channels and transporters (see below). This supports the notion that recruitment of Kir4.1 to the strial epithelium was important for the evolution of the high mammalian EP.

We note, however, that recruitment of Kir4.1 alone is insufficient to explain the high mammalian EP, as the layered structure of the cochlear lateral wall, generating an electrically isolated intrastrial space, is similarly crucial^2^,^35^. The lateral wall consists of two functional layers: (1) a syncytium of intermediate and basal cells of the stria vascularis and fibrocytes of the spiral ligament, which are connected via gap junctions, and (2) the marginal cells facing the endolymph of scala media. These two layers are separated by the intrastrial space. The EP is based on two K^+^-diffusion potentials, the first being established in the intrastrial space by Kir4.1, and maintained by Na^+^, K^+^-ATPases and NKCC1 in the basolateral membrane of the marginal cell layer, and the second one depending on KCNQ1/KCNE1 channels at the apical surface of marginal cells^2^. Thus, integration of Kir4.1 into a single layered epithelium such as the tegmentum vasculosum would be insufficient to generate a high EP in birds by this mechanism.

The role of Kir5.1, encoded by K*CNJ16*, is less clear. The gene is strongly expressed in fibrocytes of the spiral ligament^36^, which suggests an important role in K^+^ cycling in the inner ear. Yet, no hearing deficits were reported for two independent *KCNJ16* knockout mouse lines^37^,^38^. Note, however, that hearing was not explicitly tested. Its absence in the tegmentum vasculosum is consistent with the lack of a spiral ligament in birds.

In contrast to the two K^+^-channels, the other genes that are essential for K^+^ secretion in mammals, i.e. *ATP1A1*, *ATP1A2*, *ATP1A3, ATP1B1, BSND, CLCK*, *KCNQ1*, *KCNE1, SLC4A11* and *SLC12A2,* are all present in the avian tegmentum vasculosum. The molecular complement for K^+^ secretion thus appears to be highly conserved between birds and mammals. Yet, we note that our analysis lacks information on subcellular localization of these proteins, which is also important for their proper function. Enzymatic analysis of the Na^+^, K^+^ ATPase already demonstrated its expression on the basolateral membrane^39^, similar to marginal cells. It will therefore be interesting to determine the subcellular localization of the other transporters and channels as well. Due to the probable close evolutionary relationship between vestibular dark cells, marginal cells, and the cells of the tegmentum vasculosum (see below), we expect concordant subcellular localization of these proteins.

Conflicting with conflicting with our findings, the involvement of NKCC1, encoded by *SLC12A2,* in the generation of the avian endolymph was previously questioned, based on pharmacological experiments in pigeon. Application of furosemide, a blocker of NKCC1, had neither an effect on the EP nor on the spontaneous or tone-evoked discharge of single auditory-nerve fibers^18^, despite being a strong diuretic^40^. Yet, our RNA *in situ* data from both chicken and barn owl demonstrated clear expression of this gene in the avian tegmentum vasculosum. Reasons for this discrepancy remain unclear at present. It is conceivable that the intravenously applied furosemide^18^ was unable to reach its targets in the dark cells that are separated from the vascular capillaries of the tegmentum vasculosum by a basement membrane^9^. Indeed, the unique closeness of vascular capillaries and the basolateral membranes of marginal cells in the mammalian stria vascularis was suggested to be crucial for the effectiveness of intravenous administration of diuretics, because, in contrast, application of furosemide directly into scala media has little effect on the EP^41^.

We note that the tegmentum vasculosum and the marginal cells of the cochlear stria vascularis share similar expression patterns also with vestibular dark cells, the presumed secretory elements in the cristae ampullares^42^,^43^. Both the tegmentum vasculosum and the vestibular dark cells express *ATP1A1, ATP1B1, KCNQ1*, *KCNE1*, *BSDN*, *SLC12A2* and *GJB2* and *GJB6*. Except for *ATP1A2*, *ATP1A3 and ATP1B1*, all genes are expressed in the strial marginal cells as well^2^. They hence share the molecular repertoire for K^+^ secretion. This signature adds to the previously described shared cytological, molecular, and physiological properties between these three epithelia^9^,^21^,^42^. They all present with a dense cytoplasm with tightly packed mitochondria, an infolded plasma membrane at the basolateral pole, numerous microvilli at the luminal surface^9^, and Na^+^, K^+^-ATPase and Cl^−^-conductance in the basolateral membrane^21^. Additionally, K^+^ conductance is present in the apical membrane of vestibular dark cells and strial marginal cells, and K^+^ secretion is regulated via purinergic receptors, cAMP^21^, and ß1 adrenergic receptors^44^,^45^. Additionally, marginal cells and vestibular dark cells share the tight junction proteins claudin-1, -3, -8, -9, -12, -14, and -18^46^ and the expression of the atrial natriuretic peptide^47^.

These data bear important implications for the evolution of the epithelia involved in the generation of the endolymph in scala media. Collectively, the many molecular and ultrastructural similarities suggest that dark cells of the ancestral vestibular system, which arose at least 400 million years ago^48^ gave rise to those of the tegmentum vasculosum and the strial marginal cells. This is supported by their common origin from the otocyst epithelium (tegmentum vasculosum^49^, dark cells^50^, marginal cells^51^). Furthermore, dark cells of the tegmentum vasculosum and the vestibular cristae are separated from the underlying connective tissue by a basal lamina^9^,^50^,^52^, which is also present beneath the marginal cells early in development^51^.

Most of the additional cell types in the mammalian spiral ligament are also present in the vestibular epithelium, but are absent from the avian tegmentum vasculosum. Strial intermediate cells are melanocytes, which are also present in the subepithelial area of the vestibular organ. Interestingly, the same gene regulatory network module, consisting of SOX10 and the endothelin-3/EDNRB signaling pathway is involved in the generation of melanocytes in both the stria vascularis and the vestibular organ^53^. Fibrocytes, another constituent of the mammalian spiral ligament, are also present in the vestibular organ. It thus appears that the organization of these cell types into a layered system with an intrastrial space, and the recruitment of Kir4.1 to the melanocytes giving rise to the intermediate cells of the mammalian stria vascularis were crucial steps in increasing the level of the EP in mammals. The avian tegmentum vasculosum displays neither of those two crucial characteristics, yet maintains a significantly positive EP of up to +35 mV (in the owl). It remains an interesting challenge for future studies to explain how this is achieved.

### Ultimate cause: Possible selective pressures towards evolution of an increased positive EP

The selective pressure(s) resulting in the unique, highly positive EP in mammals currently remain unclear. It is commonly stated^2^ that the EP serves to increase the sensitivity of hearing by augmenting the electrical gradient as the driving force for the influx of K^+^ and Ca^2+^ during transduction. This implies that a higher EP conveys superior sensitivity. The best hearing thresholds of mammals are, however, not consistently lower than those of other vertebrates^23^. For example, an analysis of terrestrial mammals revealed an average lowest behavioral threshold of 0.4 dB SPL^54^. This is easily met by a range of bird species with behavioral thresholds close to or even below 0 dB SPL (starling -1 dB SPL, bullfinch -4.9 dB SPL, cockatiel -9.5, barn owl -18.5 dB SPL, and great horned owl -15 dB SPL^23^). If anything, the avian cochlea is thus the more sensitive, as most birds – in contrast to mammals - do not benefit from peripherally amplifying pinnae. Conversely, marsupial mammals have relatively high hearing thresholds of >20 dB SPL, despite the fact that they maintain the typically mammalian, highly positive, cochlear EP^5^. Together, these data reveal a poor correlation between hearing threshold and the EP; mammals and non-mammalian tetrapods do not divide along this line. Increased sensitivity *per se* thus cannot explain the selective pressure driving the evolution of an increased EP.

A unique feature of mammalian hearing is the extension of the upper frequency limit >10 kHz. We report here a similar correlation in the barn owl between an increased EP and extended high-frequency hearing compared to other birds. Although this does not immediately identify a mechanism, it suggests some benefit of an increased EP at higher auditory frequencies. Indeed, the EP is known to vary significantly even along the tonotopic gradient of the mammalian cochlea, from about +70 mV in the most apical, low-frequency turn to about +90 mV basally^55^.

## Conclusion

Our data shed light on the evolution of the molecular repertoire in the vertebrate inner ear. The recruitment of Kir4.1 to the mammalian inner ear was a crucial step in generating the high EP, which has no equivalent in other vertebrate groups. Furthermore, our study shows that the large-scale genomic data available for all important vertebrate groups will greatly assist in deciphering evolutionary processes in the auditory system at the molecular level. This will ultimately result in a detailed understanding of the evolution of the auditory system, which is tied to human social evolution like no other sensory system.

## Material and Methods

### Animals

Chickens (*Gallus gallus domesticus*), egg-layer breed, aged 18 to 36 days posthatching, and barn owls (*Tyto alba guttata*) aged 34 to 966 days posthatching were used. All protocols were in accordance with the German Animal Protection law and approved by local animal care and use committees (Government of Upper Bavaria and Laves, Oldenburg, Germany). Protocols also followed the NIH guide for the care and use of laboratory animals.

### Tissue fixation

Animals were injected with a lethal dose of sodium pentobarbital (Narkodorm, CP-Pharma GmbH, Burgdorf, Germany) and perfused transcardially with phosphate-buffered saline (PBS) containing (mM): 130 NaCl, 7 Na_2_HPO_4_, 3 NaH_2_PO_4_, pH 7.4, followed by fixation by 4% paraformaldehyde (PFA) in PBS. Animals were then decapitated and the head stored in PFA at 4°C overnight. For dissection of the cochlea, the middle ear cavity was opened and bony structures were removed. For brain and kidney tissue, samples were collected after perfusion of the animal and stored in 30% sucrose and 4% PFA at 4°C overnight.

### Evolutionary bioinformatical analysis

The human protein sequences of Barttin (NM_057176.2), CLC-Ka (NM_001042704.1), and CLC-Kb (NM_000085.4) were subjected to Blast analyses (Blastp, NCBI) against the databases of following organisms: *Rattus norvegicus*, *Monodelphis domestica*, *Danio rerio*, *Takifugu rubripes*, *Latimeria chalumnae*, *Xenopus (Silurana*) *tropicalis*, *Gallus gallus*, *Taeniopygia guttata*, *Anolis carolinensis*, *Strongylocentrotus purpuratus*, and *Ciona intestinalis*. Sequences with an E-value of at least 10^−2^ were saved and reverse blasted against *Homo sapiens* protein database^56^. Protein sequences that showed in the reverse blast the same Barttin, CLC-Ka and CLC-Kb protein sequences of *Homo sapiens* as a best hit were used for further analyses. The multiple sequence alignments were generated by using the default settings in MUSCLE^57^ as implemented in SeaView v4.4.2 and manually improved by eye thereafter. The phylogenetic tree of Barttin and CLC-Ka/b were constructed using maximum-likelihood analyses with a bootstrap analysis of 1,000 replicates (PhyML). The final tree was edited using FigTree.

### Immunohistochemistry

Isolated barn owl cochleae were soaked in 20% gelatin in PBS at 40 °C for 30 minutes and the block then hardened by cooling. The embedded cochlea was cut with a razorblade into three pieces which could then be oriented and cut separately. This was necessary to compensate for the pronounced curvature of the barn owl basilar papilla, in order to obtain approximate cross-sections along its full length. The individual blocks were further hardened in 4% PFA in PBS for a day and then cut at 40 μm with a vibratome (Leica VT-1000S, Wetzlar, Germany). Isolated chicken cochleae were cryprotected in 30% sucrose for a minimum of 30 minutes. They were then cut into two pieces, each of them mounted in TissueTek (VWR, Darmstadt, Germany), oriented for later cross-sectioning. Cross sections of 20 μm thickness were cut on a cryostat (CM1950, Leica Biosystems). Sections were mounted on gelatinized slides, dried and subsequently immunoreacted according to standard protocols, using 3% bovine serum albumin +0.2% TritonX-100 as blocking solution. The primary antibody was anti-Na^+^, K^+^ ATPase (Sigma-Aldrich, A276, Darmstadt, Germany) at a concentration of 1:100, coupled to a biotinylated secondary anti-mouse antibody (Vector Laboratories, BA-2000, Eching, Germany) applied at 1:100, and subsequently to CY3-labelled streptavidin (Jackson ImmunoResearch, 016-160-084, Hamburg, Germany), applied at 1:600. Control slides were treated in parallel, but omitting the primary antibody. Slides were finally coverslipped under Vectashield (Vector Laboratories) and documented using an epifluorescence microscope (Nikon Diaphot-TMD or Nikon 90i) with a digital camera attached.

### RNA Isolation and reverse transcription

Total RNA was isolated from brain and kidney of chicken by the guanidine thiocyanate method^58^. The quality and quantity of RNA samples were assessed by gel electrophoresis and optical density measurements, respectively. Reverse transcription of total RNA (10 μg) was performed using standard protocols with a mixture of random hexanucleotide and poly-T primers, and Revert Aid^TM^ M-MuLV reverse transcriptase (Thermo Scientific, St. Leon-Rot, Germany) as the enzyme^59^.

### RNA *in situ* hybridization

Probes for *ATP1A1*, *ATP1A2*, *ATP1A3*, *ATP1B2, KCNJ10*, *KCNJ16*, *KCNQ1*, *KCNE1*, *GJB2*, *GJB6*, and *SLC12A2* were generated from chicken brain cDNA, and probes for *BSND* and *CLCK* from chicken kidney cDNA. The barn owl *SLC12A2* and *KCNJ10* probes were generated from owl brain cDNA. Primers and Genbank accession numbers of the corresponding genes are given in Table 4. For barn owl *KCNJ10*, no public sequence was available and primers were therefore based on conserved regions of a multiple sequence alignment of sauropsid *KCNJ10* sequences. PCR products were cloned into the pGEM-T easy vector (Promega, Mannheim, Germany) and transcribed by T7 or SP6 polymerases in the presence of digoxigenin-11-UTP (Roche, Mannheim, Germany)^60^. Transcription by T7 polymerase leads to the generation of antisense probes, while transcription by SP6 polymerase leads to the generation of sense probes.

Cochleae were cryoprotected in 15% or 30% sucrose in PBS for a minimum of 30 minutes. They were then cut into 2 (chicken) or 4–5 (barn owl) pieces harboring approximately linear segments of basilar papilla each. The pieces were transferred to custom molds filled with TissueTek (VWR, Darmstadt, Germany), oriented for later cross-sectioning, and rapidly frozen on a metal platform cooled by liquid nitrogen. Specimens from the cochlea, brain, or kidney were stored at −80 °C until needed. Cross sections of 20 μm thickness were cut on a cryostat (CM1950, Leica Biosystems) and sections stored at −80 °C until use. On-slide *in situ* hybridization was performed at 50–60 °C overnight in hybridization buffer (50% formamide, 5 × SSC, 2% blocker (Roche), 0.02% SDS, 0.1% N-Lauryl sarcosine^60^. Bound probes were detected with an anti-Digoxigenin antibody conjugated to alkaline phosphatase (Roche). SP6 (sense) probes served as negative controls and yielded no staining. Slides were documented using DIC optics on a Nikon Eclipse 90i microsope with a digital camera attached.

### EP measurement

All electrophysiological measurements were carried out under anaesthesia, with animal homeostasis as previously described^61^,^62^. Briefly, anaesthesia was induced by intramuscular (i.m.) injection of ketamine (20 mg/kg for chickens, 10 mg/kg for owls) and xylazine (3 mg/kg). Cloacal temperature was maintained at 41.5 °C (chickens) or 39 °C (owls) and an electrocardiogram recorded to monitor anaesthesia. Anaesthesia was mostly maintained by supplementary half-doses of ketamine and xylazine or, in two chickens, by 1.5% isofluorane in carbogen, delivered via a custom respiration mask. All animals breathed unaided but most were intubated through a tracheal cut, in order to prevent complications arising from salivation. All animals also received an additional, single dose of 20 mg/kg metamizole i.m.

The head was held firmly in a custom device. In owls, an opening in the posterior skull provided a view of the columella (middle ear ossicle) and the oval and round windows of the inner ear. In chickens, the external ear canal was widened and the eardrum removed to achieve a similar view. The columella was clipped and the columellar footplate was carefully lifted from the oval window, which was then slit to open scala vestibuli and expose the tegmentum vasculosum. Borosilicate glass electrodes filled with 3 M KCl and a typical impedance of 20–50 MOhms were placed under visual control, and then advanced remotely by a precision microdrive (Burleigh inchworm 6000ULN, Burleigh Park, USA) through the tegmentum into scala media. The potential relative to a grounded Ag/AgCl pellet electrode, placed under the skin in the head region, was amplified (World Precision Instruments 767 electrometer, Berlin Germany) and fed to a National Instruments interface card (BNC 2110) mounted in a personal computer. Custom written LabView software (National Instruments) was used to continuously monitor and store the measured potential. We only accepted measurements that satisfied the following criteria: 1. Positive potential stable upon advancing the electrode a further 40–50 μm, 2. potential then stable over several minutes without moving the electrode, 3. potential returned to within a few mV of the original zero point upon retreat of the electrode. The value of the EP was taken as the potential difference on retreat (see example in Fig. 6).

At the conclusion of measurements, animals were euthanised by an overdose of sodium pentobarbital. In three chickens, this was administered while an electrode was still in scala media and the change upon death monitored.

## Additional Information

**How to cite this article**: Wilms, V. *et al.* Molecular bases of K^+^ secretory cells in the inner ear: shared and distinct features between birds and mammals. *Sci. Rep.* **6**, 34203; doi: 10.1038/srep34203 (2016).

## Figures and Tables

**Table 1:** Genbank accesion number of the amino acid sequence of CLC-K protein family members.

CLC-K protein family member	Accession number
*Homo sapiens* CLC-Ka	NP_001036169.1
*Homo sapiens* CLC-Kb	NP_000076.2
*Rattus norvegicus* CLC-K1	CAA84064.1
*Rattus norvegicus* CLC-K2	NP_775126.1
*Monodelphis domesticus* CLC-Kx1	XP_007491743.1
*Monodelphis domesticus* CLC-Kx2	XP_001365968.1
*Gallus gallus* CLC-k	XP_425749.3
*Taeniopygia gutatta* CLC-k	XP_002189185.2
*Anolis carolinensis* CLC-k	XP_008120040.1
*Latimeria chalumnae* CLC-k	XP_005996366.1
*Danio rerio* CLC-k	NP_956676.1
*Takifugu rubripes* CLC-k	XP_003963003.1

**Table 2:** Genbank accesion number of the amino acid sequence of Barttin protein family members.

Barttin protein family member	accession number
*Homo sapiens* Barttin	NP_476517.1
*Rattus norvegicus* Barttin	NP_620435.1
*Monodelphis domesticus* Barttin	XP_007480614.1
*Gallus gallus* Barttin	XP_015146557.1
*Taeniopygia gutatta* Barttin	XP_012430736.1
*Anolis carolinensis* Barttin	XP_008107597.1
*Latimeria chalumnae* Barttin	XM_014494207.1
*Xenopus tropicalis* Barttin	XP_002931511.1
*Danio rerio* Barttin	NP_001292552.1
*Takifugu rubripes* Barttin	XP_011612873.1

**Table 3:** Amino acid sequence identity between paralogous CLC-Ks across species.

	Homo sapiens CLC-Ka	Homo sapiens CLC-Kb	Rattus norvegicus CLC-K1	Rattus norvegicus CLC-K2	Monodelphis domesticus CLC-x1	Monodelphis domesticus CLC-x2
Homo sapiens CLC-Ka		91%	83%	80%	58%	71%
Homo sapiens CLC-Kb	91%		81%	81%	59%	72%
Rattus norvegicus CLC-K1	83%	81%		83%	58%	71%
Rattus norvegicus CLC-K2	80%	81%	83%		56%	70%
Monodelphis domesticus CLC-Kx1	58%	59%	58%	56%		76%
Monodelphis domesticus CLC-Kx2	71%	72%	71%	70%	76%	

**Table 4:** Primers and Genbank accession number.

Gene	Genbank accession number	Primer
gg*ATP1a1*	NM_205521	5′-GGGTAAGACTCCCATTGC-3′ 5′-CTGAAACACAGCACGGTTG-3′
gg*ATP1a2*	NM_205476	5′-AGTATTCCCCCGCTGCCACC-3′ 5′-ATGACGACAGCGGCCAGCACC-3′
gg*ATP1a3*	NM_205475	5′-TGGGGGACAAAGGGGAGAAG-3′ 5′-TGACCACGGCGGCCAAAACG-3′
gg*ATP1b1*	NM_205520	5′-TGGCCCCTAAGTATTGCTGC-3′ 5′-TAGCCTGACCCAGGTCTCTC-3′
gg*Barttin*	XM_003641691.2	5′-ATGGCCGAGGAGAAGACGTTTC-3′ 5′-CTGTGCTGATGCCTGGATCTG-3′
gg*CLC-k*	XM_425749.4	5′-GGCTTTGCCAACAGCATCAC-3′ 5′-CATGAAGGCACCACAAGTAG-3′
gg*GJB2*	XM_425641	5′-TCCTGTTCATCTTCCGTATC-3′ 5′-GTAGGCCGAGACACGAAAC-3′
gg*GJB6*	NM_204931	5′-TGTGTCCAGCATTTGCATTC-3′ 5′-TATCTGCACGAAGGCACTAG-3′
gg*KCNE1*	XR_210189	5′-CAACCTAGCCAGGTCAAAGC-3′ 5′-GCCCAAGCCAAGAAGTCAAC-3′
gg*KCNJ10*	XM_003643494	5′-CTTCATCACCGGGACCTTC-3′ 5′-AAATCCGCCACGTACTTCC-3′
gg*KCNJ16*	XM_004946241	5′-AGATGAGATGACTGTTTTGG-3′ 5′-CTCATCAGCTAGTGGTTGAC-3′
gg*KCNQ1*	XM_421022	5′-GTGGAGGACAAGGTTACGC-3′ 5′-TCTGAGCCATAATGAGTTGC-3′
gg*SLC4A11*	XM_015286072.1	5′-GAAGATTTCCAGTGTACGCG-3′ 5′-CCTTTGAAGGCATCAAGCAC-3′
gg*SLC12A2*	XM_004949378	5′-ATGGAGGGAAAGCAGCAG-3′ 5′-CTTCCTCTCCATTTGCATAGC-3′
ta*SLC12A2*	XM_009965639	5′-ATGGATTGTGGGACAAGCTG-3′ 5′-GCAAGTATGAGGATCACCAG-3′
ta*KCNJ10*	not available	5′-AGGTGTACTACAGCCAGAC-3′ 5′-CGGCGTTTTGGCTGAACTTG-3′

gg, *gallus gallus*; ta, *tyto alba*.
